# Learning the Mental Health Impact of COVID-19 in the United States With Explainable Artificial Intelligence: Observational Study

**DOI:** 10.2196/25097

**Published:** 2021-04-20

**Authors:** Indra Prakash Jha, Raghav Awasthi, Ajit Kumar, Vibhor Kumar, Tavpritesh Sethi

**Affiliations:** 1 Indraprastha Institute of Information Technology New Delhi India; 2 Adobe Noida India

**Keywords:** COVID-19, mental health, Bayesian network, machine learning, artificial intelligence, disorder, susceptibility, well-being, explainable artificial intelligence

## Abstract

**Background:**

The COVID-19 pandemic has affected the health, economic, and social fabric of many nations worldwide. Identification of individual-level susceptibility factors may help people in identifying and managing their emotional, psychological, and social well-being.

**Objective:**

This study is focused on learning a ranked list of factors that could indicate a predisposition to a mental disorder during the COVID-19 pandemic.

**Methods:**

In this study, we have used a survey of 17,764 adults in the United States from different age groups, genders, and socioeconomic statuses. Through initial statistical analysis and Bayesian network inference, we have identified key factors affecting mental health during the COVID-19 pandemic. Integrating Bayesian networks with classical machine learning approaches led to effective modeling of the level of mental health prevalence.

**Results:**

Overall, females were more stressed than males, and people in the age group 18-29 years were more vulnerable to anxiety than other age groups. Using the Bayesian network model, we found that people with a chronic mental illness were more prone to mental disorders during the COVID-19 pandemic. The new realities of working from home; homeschooling; and lack of communication with family, friends, and neighbors induces mental pressure. Financial assistance from social security helps in reducing mental stress during the COVID-19–generated economic crises. Finally, using supervised machine learning models, we predicted the most mentally vulnerable people with ~80% accuracy.

**Conclusions:**

Multiple factors such as social isolation, digital communication, and working and schooling from home were identified as factors of mental illness during the COVID-19 pandemic. Regular in-person communication with friends and family, a healthy social life, and social security were key factors, and taking care of people with a history of mental disease appears to be even more important during this time.

## Introduction

After 7 months of initial reporting, the COVID-19 pandemic continues worldwide. The mental health consequences of the COVID-19 pandemic have been substantial. More than half a million lives and more than 400 million jobs have been lost [1], causing a considerable degree of fear, worry, and concern. These effects are seen in the population at large and may be more pronounced among certain groups such as youth, frontline workers [2], caregivers, and people with chronic medical conditions. The new normal has introduced unprecedented interventions of countrywide lockdowns that are necessary to control the spread but have led to increased social isolation. Loneliness, depression, harmful alcohol and drug use, and self-harm or suicidal behavior are also expected to rise.

The Lancet Psychiatry [3] recently highlighted the needs of vulnerable groups during this time, including those with severe mental illness, learning difficulties, and neurodevelopmental disorders, as well as socially excluded groups such as prisoners, the homeless, and refugees. Calls to action for engaging more early-career psychiatrists [4,5], using technology such as telepsychiatry, and stressing the high susceptibility of frontline medical workers themselves [6] have highlighted the magnitude of the problem. Further, interventions are expected to have a gender-specific impact, with women more likely to be exposed to additional stressors related to informal care, already existing economic disparity, and school closures. Similarly, age and comorbidity status may have a direct impact on susceptibility to mental health challenges due to their relationship with COVID-19 morbidity and mortality. Indeed, it has been established that emotional distress is ubiquitous in affected populations—a finding certain to be echoed in populations affected by the COVID-19 pandemic [7]. Finally, the role of social media [8,9] is complex, with some research indicating an association between social media exposure and a higher prevalence of mental health problems [10].

However, most of these effects have been studied in isolation with a lack of modeling the collective impact of such factors. This study addresses this gap through the use of Bayesian networks (BNs), an explainable artificial intelligence approach that captures the joint multivariate distribution underlying large survey data collected across the United States. We also address the gap of vulnerability prediction for mental health events such as anxiety attacks using supervised machine learning models.

## Methods

### Data Sets

**Figure 1:**
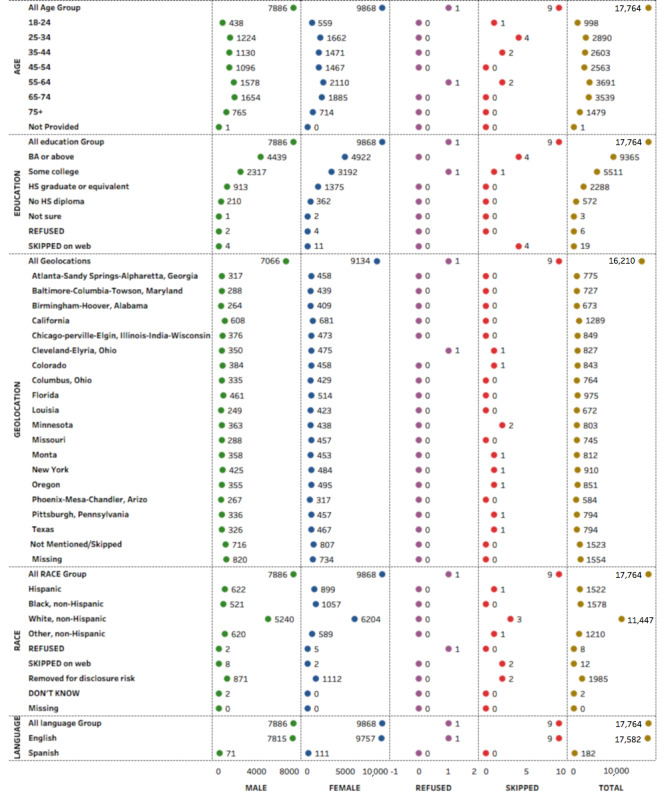
Sociodemographics of respondents who participated in the survey. It can be seen that there was almost a similar representation from both genders. Age groups 25-75 years were predominantly captured in the survey. Most of the respondents had received a Bachelor's degree or above and were nearly equally distributed across geographies within the United States. HS: high school.

We extracted the data of 17,764 adults [11] from two weekly surveys (April 20-26 and May 4-10, 2020) of the US adult household population nationwide for 18 regional areas including 10 states (California, Colorado, Florida, Louisiana, Minnesota, Missouri, Montana, New York, Oregon, Texas) and 8 metropolitan statistical areas (Atlanta, Baltimore, Birmingham, Chicago, Cleveland, Columbus, Phoenix, Pittsburgh). Two rounds of data collection were available at the time of this analysis, and both rounds of data until May 25, 2020, were included in this analysis. The details of the original data are available elsewhere [12]. To summarize, the data set comprised variables on physical health, mental health, insurance-related policy, economic security, and social dynamics. Figure 1 shows the sociodemographic characteristics of respondents participating in the survey.

### Analysis

**Figure 2:**
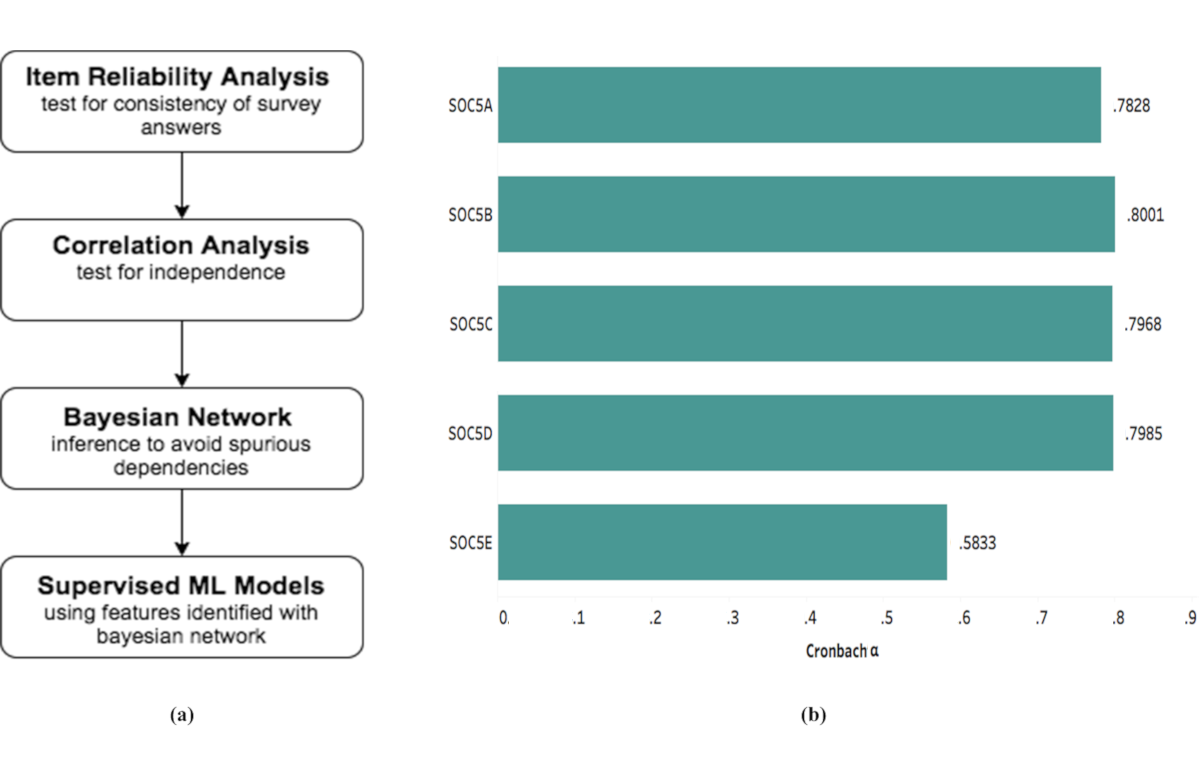
(a) Outline of the analytical pipeline. (b) Item reliability analysis of mental health indicators revealed a high degree of internal consistency (Cronbach alpha value >.70) for most of the psychological variables, thus indicating suitability for the modeling exercise. ML: machine learning.

Figure 2a shows the flow diagram for the analyses conducted. The survey questions were classified into several types of indicators such as mental health, work from home, communication, COVID-19 symptoms, chronic medical conditions, behavioral aspects, insurance assistance, and many others (Multimedia Appendix 1).

#### Item Reliability Analysis

We constructed a model for the mental health indicators with attribute *soc5a* (felt nervous, anxious*,* or on edge), attribute *soc5b* (felt depressed), attribute *soc5c* (felt lonely), attribute *soc5d* (felt hopeless about the future), and *soc5e* (sweating, trouble breathing, pounding heart, etc in the last 7 days) as outcome variables. Hence, we first evaluated the consistency in answers to the mental health questions using an item reliability analysis. A scale for measuring the reliability of internal consistency, Cronbach alpha, was calculated using the *Psych* package in R (R Foundation for Statistical Computing) [13].

#### Test of Independence Among the Mental Health Indicator and Other Indicators

Thereafter, a pairwise chi-square test of independence was performed to examine associations between *mental health indicators* and other variables, and a *P* value<.05 was taken as the cutoff for significance.

#### Data-Driven Bayesian Network Analysis

Since mental health variables may have complex dependencies with potential confounding factors, mediation, and intercausal dependency, we extended our association analysis with data-driven BN structure learning. The structure of the learned BN was made robust through bootstrapping and ensemble averaging of edge directions. The hill climbing optimizer [14] with the Akaike information criterion–based score [15] was used to select the best probabilistic graphical model that explained the data. Bootstrapped learning and majority voting over 101 BNs were done. Exact inference using the belief propagation algorithm [16] was learned to quantify the strength of learned associations. The analysis was performed in R using the package *wiseR* [17].

#### Mental Health Prediction Using Supervised Machine Learning

Next, the *Markov blanket* [18] of *mental health indicators* was extracted to select features that may predict responses to the *mental health indicators*. Data were partitioned into training (80%) and testing (20%) sets, and the class imbalance was corrected using the synthetic minority oversampling technique [19]. Different supervised machine learning models—random forest (RF), support vector machine (SVM), logistic regression, naive Bayes—were learned for predicting the response to mental health indicators using the Scikit-learn library [20] in Python.

## Results

### Item Reliability Analysis

Attribute *soc5a* (felt nervous, anxious*,* or on edge), attribute *soc5b* (felt depressed), attribute *soc5c* (felt lonely), and attribute *soc5d* (felt hopeless about the future) achieved a Cronbach alpha approximating .8 (Figure 2b), thus confirming their internal consistency and suitability for modeling.

#### Gender- and Age-Related Variation in Mental Health Indicators

**Figure 3:**
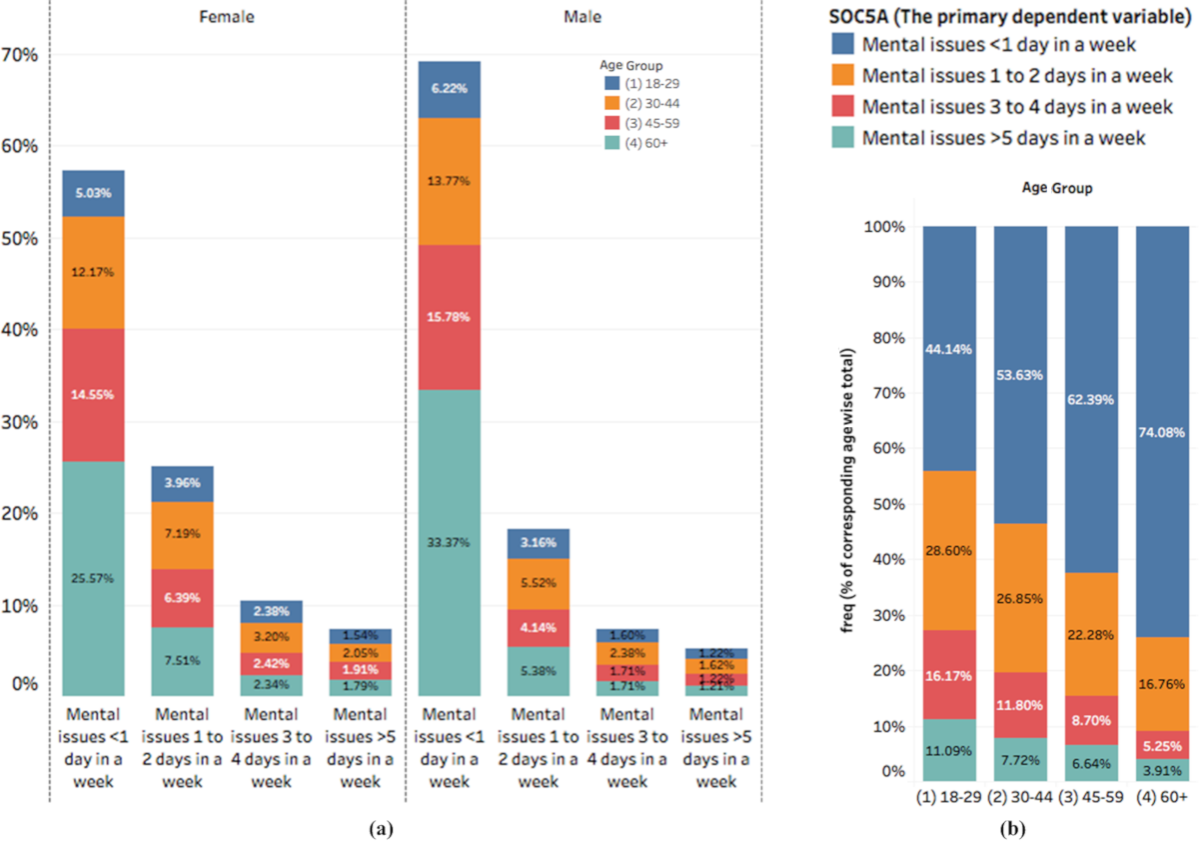
(a) Genderwise and (b) agewise distribution of mental issues (attribute *soc5a*) variable. Significance was tested using two proportion z test and chi-square test, respectively, showing a higher prevalence of mental issues among youth and in women.

Gender- and age-specific difference was observed in attribute *soc5a*, with females having a higher incidence than males (two proportion *z* test, *P*<.001; Figure 3a) and young adults in the 18-29 years age group having higher incidence than other age groups (*P*<.001; Figure 3b). The age group 18-29 years in both genders was most vulnerable to mental stress for more than 5 days in a week, thus indicating that COVID-19 may have disproportionately affected the mental health of youth due to a variety of factors.

### Associations of Anxiety in the United States

A chi-square test revealed many significant associations of the mental health variables (Multimedia Appendix 2). However, this analysis does not account for potential confounding or *explaining away* effects.

### Data-Driven Bayesian Network Analysis

**Figure 4:**
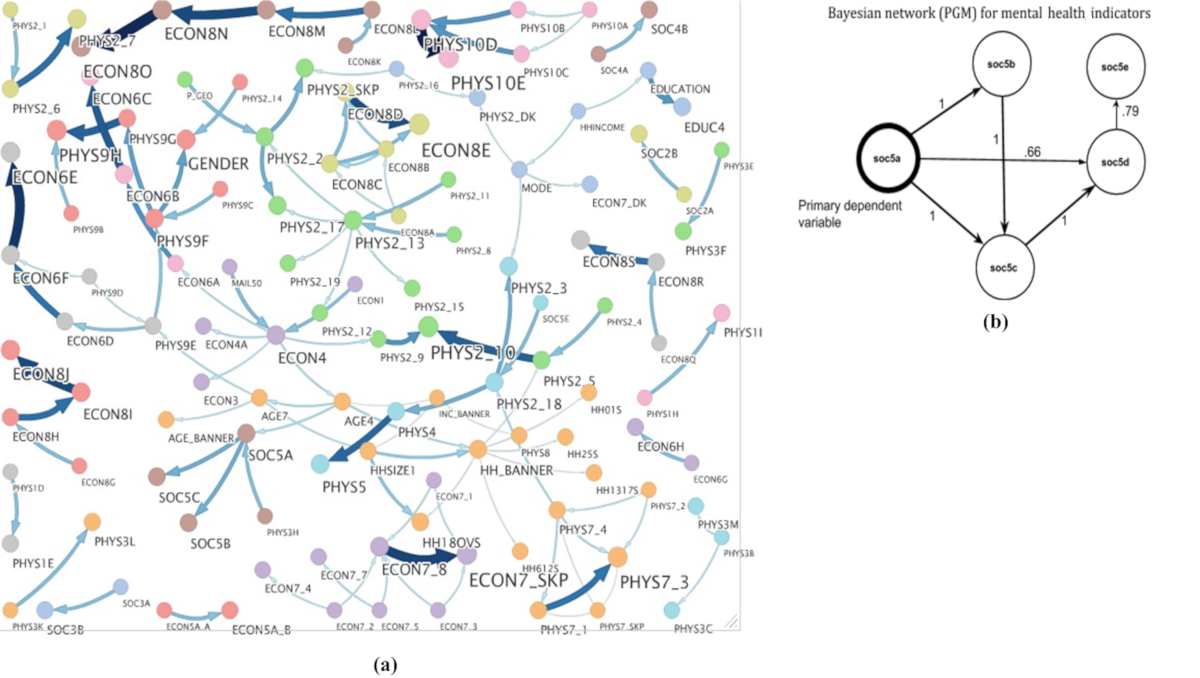
(a) Consensus structure learned through 101 bootstrapped samples. Hill climbing search along with Bayesian information criterion was used to learn the structures and connections having edge strength and direction strength more than 90% are shown. The color of the edges represents the proportion of networks in which that edge was present in the 101 bootstrapped samples, an indicator of confidence; (b) attribute *soc5a* was found to be the parent node of all other mental health variables, therefore, leading to our choice of this variable as the primary dependent variable. PGM: probabilistic graphical model.

Hence, a data-driven BN structure learning exercise was carried out and revealed interesting findings. From the learned structure, attribute *soc5a* (felt nervous, anxious, or on edge in the last 7 days) was found to be the parent variable for other mental health indicators in almost 100% of the bootstrapped networks, represented as the strength of the edges (Figure 4b). Being a driver variable in the structure, attribute *soc5a* was taken as the primary dependent variable for downstream modeling analysis.

#### Impact of Social Life and Work-Related Stressors

**Figure 5:**
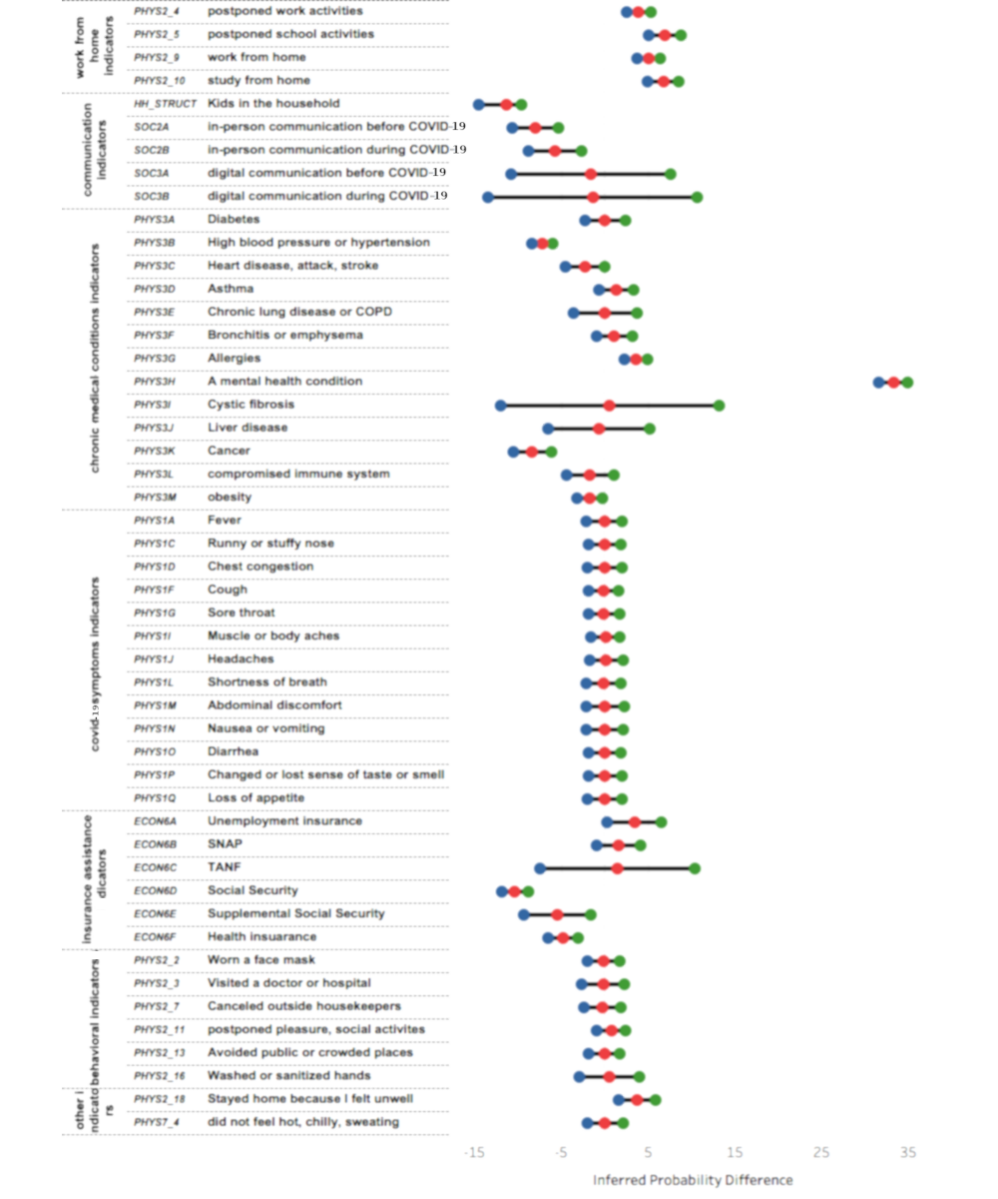
Inferences from the Bayesian network. The difference in inferred probability was calculated after conditioning the independent variables. A positive association implies a mental stress–inducing factor, whereas a negative association implies a mental stress reduction factor. The red circle shows the mean value, with green and blue showing confidence intervals. COPD: chronic obstructive pulmonary disease; SNAP: Supplemental Nutrition Assistance Program; TANF: Temporary Assistance for Needy Families.

Our analysis using network inference via the exact inference algorithm showed a clear impact of in-person social communication on the reduction of anxiety levels. A strong (>5% with CI ~1% on both sides) and (>6.5% with CI ~1.5% on both sides) monotonic increase between control of anxiety and frequency of speaking with neighbors (attribute *soc2a*, attribute *soc2b*) were observed. This effect was weaker (~1.5% with a wide confidence interval) with digital communication with friends and family conducted over phone, text, email, or other internet media (attribute *soc3a*, attribute *soc3b*). This finding underscores the importance of social communication while maintaining the appropriate measures such as masks and social distancing to maintain mental health during such isolating times. We also observed that the presence of kids in the house reduces the probability of depression by >11% with CI ~2% on both sides. Furthermore, the exact inference upon the network revealed an increase in the conditional probability of anxiety (attribute *soc5a*) arising from canceled or postponed work (>4% with CI ~1.4%), canceled or postponed school (7% with CI ~1.5%), working from home (>5% with CI ~1.3%), and studying from home (>7% with CI ~1.8%). Interestingly, although 83% of all volunteers chose to wear the mask, 77% avoided restaurants, and 83% avoided public and crowded places, these measures were not found to be associated with a significant change in anxiety levels as inferred from our model. These inferences are summarized in Figure 5.

#### Impact of Symptoms and Comorbidities

We also investigated the relationship between mental stress and COVID-19 symptoms indicators. The World Health Organization recommends contacting health service providers if any COVID-19 symptoms (attributes *phys1a* to *phys1q*) are experienced within the last 7 days. Our network did not indicate any significant impact of these responses on mental health (attribute *soc5a*), the conditional probability of which remained unchanged (62.2%) across the responses. Although medical conditions (attributes *phys3a* to *phys3m*) are known to increase the risk of serious illness from COVID-19, our model showed that having cancer (attribute *phys3k*) and hypertension (attribute *phys3b*) had a reverse impact on anxiety levels. Those with cancer had approximately 8.3% (with ~2% CI) higher conditional probability of having less than 1 anxiety-ridden day in a week (>7% effect for hypertension with CI ~1.5%). Additionally, cystic fibrosis (attribute *phys3i*) and liver disease (attribute *phys3j*) had wide confidence intervals with nonsignificant differences in mean values (Figure 5).

#### Impact of Economic Factors

Receiving income assistance through Social Security improved the conditional probability of less than 1 day of anxiety in a week by 10.4% (with CI ~1.5%) as compared with the segment of people who did not apply or receive it. Just applying for income assistance led to a 4% improvement (Figure 5). Supplemental Social Security (~5.5% with CI ~4%) and health insurance (~5% with CI ~2%) also led to similar results.

In addition to this, older adults (>60 years) found health insurance more relaxing than younger people. COVID-19 has also severely affected the financial condition of individuals, which may also lead to mental stress.

### Predictive Modeling for Susceptibility to Anxiety Attacks

Our supervised modeling approach used the Markov blanket of the attribute *soc5a* variable, that is age (attribute *age4),* physical symptoms in the last 7 days (attribute *phys7_4*)*,* staying at home (attribute *phys2_18*), and prior clinical diagnosis of any mental health condition (attribute *phys3h*) as predictors.

The following three prediction scenarios were considered:

1. Mental issues *less than 1 day* in a week (class 1) versus mental issues *more than 1 day* in a week (class 0)
2. Mental issues *less than 1 day* in a week (class 1) versus mental issues *more than 1 day* in a week (class 0)
3. Mental issues *less than 1 day* in a week (class 1) versus mental issues *more than 1 day* in a week (class 0)

RF models achieved the best performance in comparison with SVM, logistic regression, and naive Bayes models on the basis of standard model performance indicators (accuracy, sensitivity, specificity, area under the receiver operating characteristic curve; summarized in Table 1). We observed a decay (accuracy from 0.80 to 0.64; Table 1) in model predictability as we moved from high risk of depression (case 3) to low risk of depression (case 1; Table 1). Such a trend was visible with all four machine learning techniques we used.

**Table 1:** Model performance indicators of the supervised model for prediction of stress.

Scenarios			Random forest		Support vector machine		Naive Bayes		Logistic regression
**Mental issues less than 1 day in a week (class 1) vs mental issues more than 5 days in a week (class 0)**									
	Accuracy (±CI)	0.80 (0.016)		0.80 (0.016)		0.77 (0.017)		0.77 (0.017)	
	Sensitivity (±CI)	0.59 (0.063)		0.56 (0.063)		0.59 (0.063)		0.59 (0.063)	
	Specificity (±CI)	0.82 (0.016)		0.82 (0.016)		0.79 (0.017)		0.78 (0.017)	
	AUROC^a^ (±CI)	0.71 (0.026)		0.69 (0.026)		0.69 (0.025)		0.68 (0.025)	
**Mental issues less than 1 day in a week (class 1) vs mental issues more than 3 days in a week (class 0)**									
	Accuracy (±CI)	0.72 (0.018)		0.72 (0.018)		0.74 (0.017)		0.73 (0.018)	
	Sensitivity (±CI)	0.6 (0.041)		0.6 (0.041)		0.56 (0.041)		0.57 (0.041)	
	Specificity (±CI)	0.75 (0.018)		0.75 (0.018)		0.78 (0.017)		0.76 (0.018)	
	AUROC (±CI)	0.68 (0.022)		0.67 (0.022)		0.67 (0.022)		0.67 (0.022)	
**Mental issues less than 1 day in a week (class 1) vs mental issues more than 1 day in a week (class 0)**									
	Accuracy (±CI)	0.66 (0.019)		0.66 (0.019)		0.65 (0.019)		0.62 (0.019)	
	Sensitivity (±CI)	0.48 (0.027)		0.49 (0.027)		0.45 (0.026)		0.61 (0.026)	
	Specificity (±CI)	0.77 (0.018)		0.76 (0.018)		0.77 (0.018)		0.64 (0.020)	
	AUROC (±CI)	0.62 (0.019)		0.62 (0.019)		0.61 (0.020)		0.62 (0.018)	

^a^AUROC: area under the receiver operating characteristic curve.

## Discussion

Mental health is a public health concern. Mood disorders and suicide-related outcomes have increased substantially over the last decade among all age groups and genders [21,22]. The rapid spread of COVID-19 forced governments worldwide to close public gathering places, schools, colleges, restaurants, and industries. Social isolation, digital communication, and working and schooling from home have become the new normal, and many jobs have been lost. Collectively, this has triggered a high level of anxiety, stress, and depression globally. We did not find studies that have used models to not only predict but also explain the subtle effects of life situations on mental health. An explainable probabilistic graphical modeling approach with bootstraps and exact inference allowed us to capture many of these effects in a robust manner. Our study revealed that individuals with a prior diagnosis of any mental illness are the most vulnerable for mental illness during the COVID-19 pandemic, which recommends building national-level policies to regularly track their mental status and treat them accordingly. Most importantly, our results reiterate the economic underpinnings of a collective mental health response. Income assistance via Social Security or Supplemental Social Security had a demonstrable effect on the alleviation of anxiety as inferred from our model, which provides the first scientific evidence, to the best of our knowledge, proving the utility of such efforts. The extent of such measures’ effect may be captured in such modeling studies conducted in various parts of the world, with widely varying assistance structures during this time.

Our findings from the United States can also stimulate further cultural and social research in other geographies with similar or different social structures. For example, the effects of in-person communication, as opposed to digital connectedness, may be different in countries where community living and joint families are still commonplace, such as India. Digital connectedness was not as effective as talking to a neighbor, at least in the United States, highlighting that these have fundamentally different influences on mental health and need to be further explored in systematic studies. We conjecture that such differences may arise from the evolutionary mechanisms that have shaped human societies to live and share in close physical connectedness. Such an effect has been previously shown in primates kept in isolation who display depressive symptoms [23,24]. Similarly, parenting and its association with neuropeptide hormones may partially explain [25] our results that the presence of kids reduces anxiety levels. Interestingly, the COVID-19 pandemic has created a unique natural experiment on the collective mental health response of individuals to a health emergency.

The life cycle of such a response may need to be further studied as the world goes through various phases of the pandemic until its resolution. However, our study indicates that the mental health impact is observable within a span of a few months, especially on young individuals. Further research will be needed, ideally in a longitudinal setting, where the same individuals can be surveyed again to understand the dynamics of the collective mental health response.

Our results also highlight that modern technological development in virtual communication is not able to replace natural socializing. Hence, it becomes imperative to design better and more empathetic technological tools that may shape a society and prevent isolation and alienation even while maintaining physical distancing and preventive measures for limiting spread. Personalization and contextualization of such measures will also be important, as our results indicate that persons with previous mental health conditions may be disproportionately affected.

Finally, our results indicate that it may be possible to identify people at the highest risk of developing mental health disturbances. Our model achieved its best performance for those who were most vulnerable (having mental stress more than 5 days in a week) versus least vulnerable (having no stress or less than 1 day of stress in a week). This can help in the segmentation of vulnerable populations such as frontline health care workers and those who are facing disproportionately higher levels of stress during this time.

A key factor in clinical and public health models is transparency and explainability in the face of complex interactions. Mental health variables are expected to have complex dependencies with potential confounding factors, mediation, and intercausal dependency; therefore, we extended our association analysis with data-driven structure learning of a BN. We preferred this approach over black box machine learning and standard statistical modeling for several reasons. Structure learning allows us to discover and model confounding factors transparently, whereas black box machine learning models such as RFs and gradient boosted machines are not well suited for transparent reasoning. Standard statistical approaches make it humanly impossible to model interactions among hundreds of variables. Structure learning allows discovery and dissection of interactions into mediation, confounding, and intercausal effects. The challenge of incorrect learning is addressed by ensembling many BNs (101 in our case) and choosing the ensemble voted structure. Our artificial intelligence (AI) approach has earlier been validated for public health problems [26,27], and this study demonstrates the underexplored potential of such an approach in complex mental health scenarios.

Our study has a few limitations. Establishing causal inference in cross-sectional data is nearly impossible, and we acknowledge the possibility of confounders. However, this was precisely the reason we chose the structure learning approach, as some of the confounding influences can be transparently discovered and explained. The ensemble voted structure over the sufficiently large number of bootstrapped structures is expected to be robust, as a set of 101 BNs was found to be sufficiently large enough for this study to address the challenge of incorrect learning. Our approach is best suited as a probabilistic reasoning model to explain mental health determinants and to make predictions, a useful outcome in COVID-19–induced mental health morbidity. We could not explain why anxiety levels may be lower in persons with pre-existing cancer or hypertension. This may be a result of reduced work environment–related stress or more contact with family members at home. However, the current data set is not suited to address this at a finer level of explainability. In addition, we could not comment upon the temporality and persistence of these effects. Our results are currently limited to only one geography (ie, the United States). However, the relatively large sample size and multiethnic involvement in the survey makes the model representative for most of the ethnicities and influences across the United States; hence, it is likely to hold true in the United States. Finally, we believe that our study contributes to the use of explainable AI to predict mental health at a population level using survey data, hence making it broadly applicable. Survey data sets are notoriously noisy, and our approach achieved a balance between knowledge discovery and a predictive accuracy of 80%, thus establishing a baseline under a novel scenario. Our algorithms can be used as a screening method for identifying individuals who need help, and further studies with additional measurements and features may increase the accuracy of predictions. Therefore, predictive models for screening and assessing the mental health impact of COVID-19 is a crucial step toward proactive management and prevention of psychiatric comorbidities as populations continue to fight the pandemic.

## Appendix

Multimedia Appendix 1 Variable groups as indicators.

Multimedia Appendix 2 Supplementary figures.

## Abbreviations

BN: Bayesian network
RF: random forest
SVM: support vector machine
