# Supplementary material for: Learning the Mental Health Impact of COVID-19 in the United States With Explainable Artificial Intelligence: Observational Study
Source: JMIR Ment Health. 2021 Apr 20;8(4):e25097. doi: 10.2196/25097 (PMC8059787; doi:10.2196/25097)
Supplement: Multimedia Appendix 2 [file mental_v8i4e25097_app2.pdf]

Supplementary material

Heatmap of negative log P values

	SOC5A	SOC5B	SOC5C	SOC5D	SOC5E
PHYS1A	6.000	6.000	6.000	6.000	6.000
PHYS1B	6.000	6.000	6.000	6.000	6.000
PHYS1C	6.000	6.000	6.000	6.000	6.000
PHYS1D	6.000	6.000	6.000	6.000	6.000
PHYS1E	6.000	6.000	6.000	6.000	6.000
PHYS1F	6.000	6.000	6.000	6.000	6.000
PHYS1G	6.000	6.000	6.000	6.000	6.000
PHYS1H	6.000	6.000	6.000	6.000	6.000
PHYS1I	6.000	6.000	6.000	6.000	6.000
PHYS1J	6.000	6.000	6.000	6.000	6.000
PHYS1K	6.000	6.000	6.000	6.000	6.000
PHYS1L	6.000	6.000	6.000	6.000	6.000
PHYS1M	6.000	6.000	6.000	6.000	6.000
PHYS1N	6.000	6.000	6.000	6.000	6.000
PHYS1O	6.000	6.000	6.000	6.000	6.000
PHYS1P	6.000	6.000	6.000	6.000	6.000
PHYS1Q	6.000	6.000	6.000	6.000	6.000
PHYS2_1	3.929	5.110	4.370	3.732	5.999
PHYS2_2	5.834	3.518	5.701	5.602	5.999
PHYS2_3	4.527	4.797	3.146	5.810	6.000
PHYS2_4	6.000	6.000	6.000	6.000	6.000
PHYS2_5	6.000	6.000	6.000	6.000	6.000
PHYS2_6	5.999	6.000	6.000	6.000	5.978
PHYS2_7	0.986	3.797	2.125	2.636	1.698
PHYS2_8	6.000	6.000	6.000	6.000	5.999
PHYS2_9	6.000	6.000	6.000	6.000	5.420
PHYS2_10	6.000	6.000	6.000	6.000	6.000
PHYS2_11	6.000	6.000	6.000	6.000	6.000
PHYS2_12	6.000	6.000	6.000	6.000	6.000
PHYS2_13	6.000	6.000	6.000	6.000	6.000
PHYS2_14	1.086	1.349	3.591	1.484	0.768
PHYS2_15	6.000	6.000	6.000	6.000	6.000
PHYS2_16	2.699	3.970	2.889	3.165	2.324
PHYS2_17	5.772	1.669	2.388	2.563	0.668
PHYS2_18	6.000	6.000	6.000	6.000	6.000
PHYS2_19	4.722	3.945	5.556	3.338	6.000
ECON6A	6.000	6.000	6.000	6.000	6.000
ECON6B	6.000	6.000	6.000	6.000	6.000
ECON6C	5.973	6.000	5.999	6.000	6.000
ECON6D	6.000	6.000	6.000	6.000	6.000
ECON6E	6.000	6.000	6.000	6.000	6.000
ECON6F	6.000	6.000	6.000	6.000	6.000
ECON6G	6.000	6.000	6.000	6.000	6.000
PHYS3A	6.000	6.000	6.000	5.951	6.000
PHYS3B	6.000	6.000	6.000	6.000	5.993
PHYS3C	6.000	6.000	6.000	5.979	5.999
PHYS3D	6.000	6.000	6.000	6.000	6.000
PHYS3E	6.000	6.000	6.000	4.895	6.000
PHYS3F	6.000	6.000	6.000	6.000	6.000
PHYS3G	6.000	6.000	6.000	6.000	6.000
PHYS3H	6.000	6.000	6.000	6.000	6.000
PHYS3I	6.000	6.000	4.516	2.490	6.000
PHYS3J	6.000	6.000	5.777	5.756	6.000
PHYS3K	6.000	6.000	6.000	5.999	6.000
PHYS3L	6.000	6.000	6.000	6.000	6.000
PHYS3M	6.000	6.000	6.000	6.000	6.000
SOC2A	6.000	6.000	6.000	6.000	5.671
SOC2B	6.000	6.000	6.000	6.000	5.972
SOC3A	5.847	6.000	6.000	3.953	2.732
SOC3B	5.955	5.835	6.000	4.156	1.039

F3

0.668 6.000

Sum of F3 broken down by F2 vs. F1. Color shows sum of F3. The marks are labeled by sum of F3.

Supplementary Figure 1: Heatmap for negative log p values of associations.

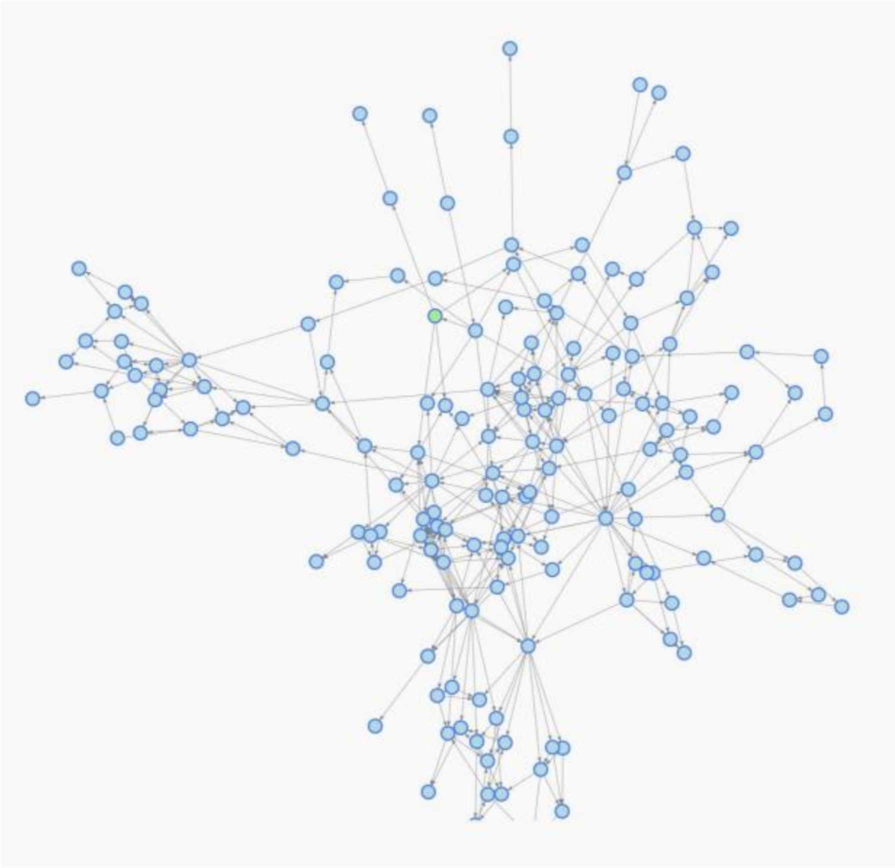

Supplementary Figure 2: Bayesian Network topology

Supplementary Table 1: Description of attributes in survey data-set.

Code	Attributes Description
**mental_health_indicators**<br>*(questions related to mental stress in last seven days)*	
soc5a	Felt nervous, anxious, or on edge
soc5b	Felt depressed
soc5c	Felt lonely
soc5d	Felt hopeless about the future
soc5e	sweating, trouble breathing, pounding heart, etc
**work_from_home_indicators**<br>*(questions related to work and school from home)*	
phys2_9	work from home
phys2_10	study from home
phys2_4	postponed work activities
phys2_5	postponed school activities
**communication_indicators**<br>*(questions related to communication with friends/neigbhour/family)*	
soc2a	in-person communication during COVID
soc2b	in-person communication before COVID
soc3a	digital communication during COVID
soc3b	digital communication before COVID
**covid_symptoms_indicators**<br>*(questions on covid symptoms Prescribed by WHO)*	
phys1a	Fever
phys1b	Chills
phys1c	Runny or stuffy nose
phys1d	Chest congestion
phys1e	Skin rash
phys1f	Cough
phys1g	Sore throat
phys1h	Sneezing
phys1i	Muscle or body aches
phys1j	Headaches
phys1k	Fatigue or tiredness
phys1l	Shortness of breath
phys1m	Abdominal discomfort
phys1n	Nausea or vomiting
phys1o	Diarrhea
phys1p	Changed or lost sense of taste or smell
phys1q	Loss of appetite
**clinical_history_indicators**<br>*(questions on prior clinical history of diseases)*	

phys3a	Diabetes
phys3b	High blood pressure or hypertension
phys3c	Heart disease, attack, stroke
phys3d	Asthma
phys3e	Chronic lung disease or COPD
phys3f	Bronchitis or emphysema
phys3g	Allergies
phys3h	A mental health condition
phys3i	Cystic fibrosis
phys3j	Liver disease
phys3k	Cancer
phys3l	compromised immune system
phys3m	obesity
**behavioural_indicators** *(questions on behavioral changes propsed/enforced by government)*	
phys2_1	Canceled a doctor appointment
phys2_2	Worn a face mask
phys2_3	Visited a doctor or hospital
phys2_7	Canceled outside housekeepers
phys2_8	Avoided restaurants
phys2_11	postponed pleasure, social activites
phys2_12	Stockpiled food or water
phys2_13	Avoided public or crowded places
phys2_16	Washed or sanitized hands
**insurance_assistance_indicators** *(questions on insurance and different assistance)*	
econ6a	Unemployment insurance
econ6b	SNAP
econ6c	TANF
econ6d	Social Security
econ6e	Supplemental Social Security
econ6f	Health insuarance
econ6g	aid from the government
**Other_useful_Indicators**	
phys7_4	did not fell hot, chilly, sweating
phys2_18	Stayed home because I felt unwell
